# Ligand Binding Mechanism and Its Relationship with Conformational Changes in Adenine Riboswitch

**DOI:** 10.3390/ijms21061926

**Published:** 2020-03-11

**Authors:** Guodong Hu, Haiyan Li, Shicai Xu, Jihua Wang

**Affiliations:** Shandong Key Laboratory of Biophysics, Dezhou University, Dezhou 253023, China; tianwaifeixian78@163.com (H.L.); shicaixu@dzu.edu.cn (S.X.)

**Keywords:** adenine riboswitch, molecular dynamics simulation, ligand binding mechanism, relationship between ligand binding and bio-function

## Abstract

Riboswitches are naturally occurring RNA aptamers that control the expression of essential bacterial genes by binding to specific small molecules. The binding with both high affinity and specificity induces conformational changes. Thus, riboswitches were proposed as a possible molecular target for developing antibiotics and chemical tools. The adenine riboswitch can bind not only to purine analogues but also to pyrimidine analogues. Here, long molecular dynamics (MD) simulations and molecular mechanics Poisson–Boltzmann surface area (MM-PBSA) computational methodologies were carried out to show the differences in the binding model and the conformational changes upon five ligands binding. The binding free energies of the guanine riboswitch aptamer with C74U mutation complexes were compared to the binding free energies of the adenine riboswitch (AR) aptamer complexes. The calculated results are in agreement with the experimental data. The differences for the same ligand binding to two different aptamers are related to the electrostatic contribution. Binding dynamical analysis suggests a flexible binding pocket for the pyrimidine ligand in comparison with the purine ligand. The 18 μs of MD simulations in total indicate that both ligand-unbound and ligand-bound aptamers transfer their conformation between open and closed states. The ligand binding obviously affects the conformational change. The conformational states of the aptamer are associated with the distance between the mass center of two key nucleotides (U51 and A52) and the mass center of the other two key nucleotides (C74 and C75). The results suggest that the dynamical character of the binding pocket would affect its biofunction. To design new ligands of the adenine riboswitch, it is recommended to consider the binding affinities of the ligand and the conformational change of the ligand binding pocket.

## 1. Introduction

Riboswitches are noncoding RNAs that function as genetic switches and they are mostly found in bacteria [1,2]. They are located in the 5′ untranslated region of messenger RNAs (mRNAs) and consist of two domains: a conserved aptamer domain that forms a binding pocket to bind metabolite small molecules, and an expression platform that controls the expression of the downstream gene(s). Generally, ligand binding to the aptamer domain allosterically alters specific structural elements of the joining expression platform, which modulates the expression of downstream genes [3,4]. Thus, riboswitches were proposed as a novel class of molecular target for developing antibiotics and chemical tools [5]. 

Riboswitches normally bind their ligands with high selectivity and affinity and often reject their cognate ligand seven when they are close chemical relatives [6]. Riboswitches can bind to primary metabolites of a cell [7], which include nucleobases (adenine and guanine) [8,9], amino acids (glycine and lysine) [10,11], cofactors such as flavin mononucleotide [12], *S*-adenosylhomocysteine, adenosylcobalamin, *S*-adenosylmethionine, cobalamine, and thiamine pyrophosphate [13], and metal ions such as Mg^2+^ [14]. 

**Figure 1:**
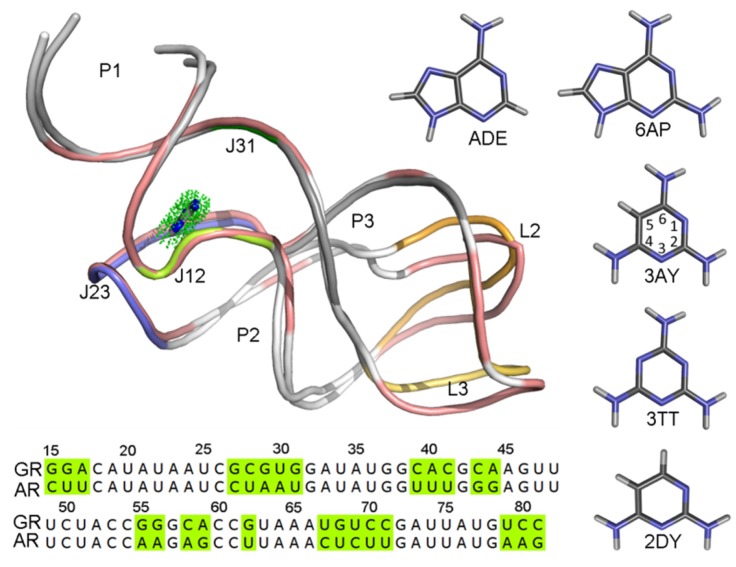
The superimposition of structures and comparison of sequences for the guanine (GR) and adenine riboswitches (AR). The structures of riboswitches are shown in cartoon representation. The structures with different sequences of two purine riboswitches are colored in salmon color for GR, AR is colored with each secondary structure and labeled. The ligand in the binding pocket is shown using a stick-and-dot representation. Five ligands are also shown in stick representation.

Purine riboswitches were identified in 2003, which contain guanine [8] and adenine [9] classes. In spite of the different sequence with a 60% identity [15] and binding different metabolites, their aptamer domains have a high structural similarity with a root-mean-square deviation (RMSD) of 1.7 Å via superposition of the phosphates (Figure 1) [16], and they are remarkable for the variety in their strategy for gene expression control and for their ligand selectivity. The *add* adenine riboswitch (AR) controls gene expression via transcriptional activation, whereas the *xpt*-pbuE guanine riboswitch (GR) controls gene expression through the termination of transcription. The crystallographic structures revealed that the aptamer domains of guanine and adenine are three-way junctions formed by three helices, connected by three junction loops, and capped with two loops [8,9]. 

The ligand binding pocket is located at the center of the three-way junction structure. Only one nucleotide is different in their binding pockets, but the selectivity of purine riboswitches is striking [17]. A Watson–Crick base pair formed between ligand and C74 for guanine versus U74 for the adenine affects the selectivity. Experimental and theoretical studies showed that purine riboswitches are capable of binding not only to purine analogues but also to pyrimidine analogues, which is accomplished by the inherent flexibility of riboswitches [18,19]. The ligands are almost completely buried with only 5% of the surface still solvent-accessible [20], and they interact with key nucleotides via aromatic stacking and hydrogen bonding (H-bond), suggesting an extensive induced-fit binding mechanism. Based on the structural similarity and the ligand selectivity for guanine and adenine riboswitches, some groups studied the mutation of C74 to U74 in guanine riboswitches, named GR(C74U), instead of adenine riboswitches [3,16,17,19].

Aqvist et al. analyzed the energetics of ligand binding to purine riboswitches by employing molecular dynamics simulations, free energy perturbation calculations, and the linear interaction energy method, explaining how the functional groups of ligands affect the binding model and affinity [3]. Batey et al. investigated the interaction of purine riboswitches with purine analogues with varying functional groups at the 2- and 6-positions [19]. Our group also studied the ligand binding mechanism of four guanine analogue complexes with guanine and adenine riboswitches [17]. Liu’s group studied the interactions and dynamic behavior of GR with a ligand using molecular docking and molecular dynamics simulations [21,22]. A study by Batey et al. showed that pyrimidine analogues can efficiently bind to purine riboswitches. Several ligands of pyrimidine analogues bear amino groups at the 5- and 6-position which may mimic the two nitrogen atoms of purines [16]. A comparison between pyrimidine analogues and purine analogues would be helpful to provide new ligand designing strategies. 

Purine riboswitches change their conformation by binding with a conjugate ligand to control the gene expression. Extensive experimental data suggest that conformational dynamics plays an important role in ligand binding and recognition at multiple levels [23,24]. Single-molecule fluorescence resonance energy transfer spectroscopy was employed to study the conformational dynamics of the *xpt* GR aptamer, suggesting a time scale of seconds for ligand binding and dissociation [25,26,27]. Hence, arranging the conformation of a riboswitch is a very slow process [6]. Wang et al. demonstrated the course of binding between the adenine riboswitch and a ligand involving two apo states, one ligand-bound intermediate, and a final bound state by applying femtosecond X-ray free electron laser (XFEL) pulses [28]. 

Molecular dynamics (MD) simulations can provide dynamical and structural characteristics at the atomic level [29]. Conformational changes of the aptamer domain in both ligand-bound and ligand-unbound states for the *S*-adenosylmethionine-, adenine-, and guanine-sensing riboswitches were investigated using MD simulations [30,31,32]. MD simulations combined with the binding free energy calculation would be more useful for obtaining the ligand binding and selectivity mechanism [6,33,34]. The inhibitor binding affinities for not only protein and ligand complexes [35,36,37,38] but also RNA and ligand complexes [3,39,40,41,42] were successfully estimated via many computational methods with varying levels of computational expense and accuracy. These methods include free energy perturbation (FEP) [43], thermodynamic integration, umbrella sampling (US) [44], and molecular mechanics Poisson–Boltzmann surface area (MM-PBSA) [45]. The MM-PBSA method combines molecular mechanics (MM) energies and entropic contributions with continuum solvent models [46,47] that can rank the binding affinity of a series of ligands targeting RNA [17,48].

In this work, the binding mechanisms of five ligands complexed with GR(C74U) and AR were studied. Figure 1 shows the structures of the five ligands. The binding free energy for each complex was calculated using the MM-PBSA method with 5000 snapshots extracted from 10 unbiased MD simulations for 100 ns each. The calculated binding free energies provide useful information for the drug–target relationship. The dynamical behaviors in GR(C74U) and AR in the adenine (ADE) bound and unbound states were investigated based on 8 μs of MD simulations in total. The results provide valuable information for understanding the conformational changes caused by ligand binding.

## 2. Results and Discussion

### 2.1. Analysis of Binding Free Energies 

The absolute binding free energies for all complexes of GR(C74U) and AR were calculated using the mm_pbsa program in AMBER 14 (Table 1). Table 1 also shows the experimental data of the seven complexes. As shown in Table 1, the predicted absolute binding free energies of the seven complexes are in agreement with their experimental results with a coefficient of determination $r^{2}$ of 0.95. 

The predicted binding free energies of 2DY are −4.58 and −4.41 kcal/mol in the GR(C74U) and AR complexes, respectively, which are much larger as compared to the others. This is also in agreement with the experimental data (Table 1) [16,26]. The total binding free energy was decomposed into individual energies in the MM-PBSA method, which is helpful when evaluating the binding mechanism of a complex in detail [49,50,51]. The sum of van der Waals interactions and nonpolar solvation energies ($\Delta G_{\mathrm{vdw}+\mathrm{nonpol}}$) was favorable for all complexes, which was responsible for the burial of the inhibitor’s hydrophobic groups upon binding. The electrostatic contribution ($\Delta G_{\mathrm{ele}+\mathrm{pol}}$) was unfavorable in all complexes except in the 6AP and AR complex. The entropic contributions (−TΔS) were unfavorable for all complexes. As shown in Table 1, the experimental binding affinities suggested that three ligands (ADE, 6AP, and 3AY) are able to bond more strongly to AR than GR(C74U). The predicted binding affinity of 3TT was larger in AR than GR(C74U). To further explore these results, the energetic terms between complexes of GR(C74U) and AR were compared, and it was found that the differences in total binding free energies ($\Delta G_{\mathrm{bind}}$) resulted mainly from the electrostatic contribution ($\Delta G_{\mathrm{ele}+\mathrm{pol}}$).

### 2.2. The Key Nucleotides for the Binding to Ligands

**Figure 2:**
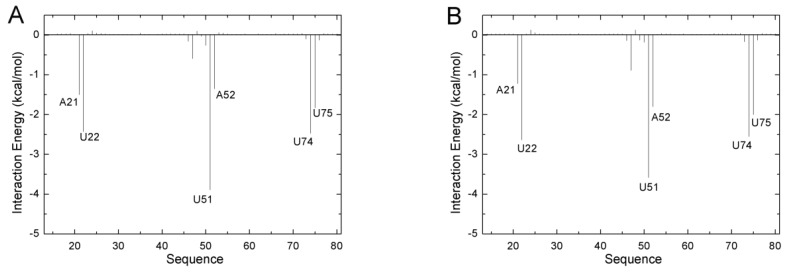
The decomposition of inhibitors on a per-nucleotide basis for the ADE and GR(C74U) complex (**A**), as well as for the ADE and AR complex (**B**).

In order to evaluate the differences in binding mechanism between both aptamers, the contribution of each nucleotide to the total binding free energy was calculated based on 5000 snapshots obtained from 10 unbiased simulations from the last 50 ns of the MD trajectories with an interval of 100 ps by utilizing the free energy decomposition method. Figure 2 shows the results of the ADE complexes, and Figure S1 (Supplementary Materials) shows the results for the complexes of the other four ligands. The contribution of a nucleotide is divided into four terms (the electrostatic interaction energy, van der Waals energy, polar solvation contribution, and nonpolar solvation contribution). The polar solvation contribution was calculated with the generalized Born solvation model, and the same parameters as the total binding free energy were applied to calculate the other three components. Several donor–acceptor systems successfully applied this method to investigate the binding mechanism [37,52].The interactions mainly involved six nucleotides (A21, U22, U51, A52, U74, and U75) with a favorable energy less than −1.0 kcal/mol in most complexes (Figure 2 and Figure S1, Supplementary Materials). These six key nucleotides can be divided into two groups. One group (A21, U22, A52, and U75) was driven by van der Waals energy, mostly due to aromatic stacking. The other (U51 and U74) group gave a large electrostatic contribution. Although these ligands are neutral, they have polar groups or atoms, which form H-bonds with the polar groups or atoms of RNA. Based on comparisons between the spectra of GR(C74U) and AR, we noted no large difference (Figure 2 and Figure S1, Supplementary Materials). The binding model of GR(C74U) is the same as that of AR. The crystallographic structures reveal that the nucleotides around the binding pocket in GR(C74U) are the same as those in AR (Figure 1). Thus, the guanine riboswitch with C74U mutated can mimic the adenine riboswitch.

### 2.3. Comparison between ADE and 6AP Complexes

**Figure 3:**
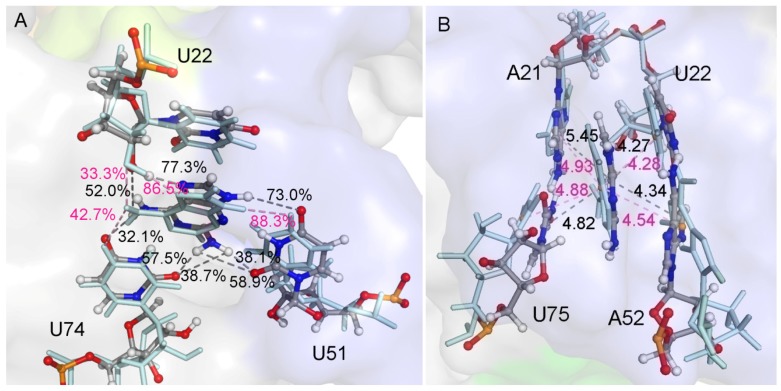
(**A**) Superimposition of two representative structures of GR(C74U) complexed with ADE and 6AP based on three nucleotides. The occupancies of hydrogen bonds (H-bonds) formed between three nucleotides and ligands are labeled. (**B**) Superimposition of two representative structures of GR(C74U) complexed with ADE and 6AP based on four nucleotides which interacted with ligands via a van der Waals interaction. The van der Waals interactions are labeled with distances. The ligands and nucleotides in the ADE complex are shown in a pale cyan color with stick representation. In the 6AP complex, they are shown using a stick-and-ball representation colored by elemental name. The labels in ADE and 6AP complexes are shown in pink and black colors, respectively.

A hydrogen atom at the 2-position in ADE is replaced with an amidogen in 6AP (Figure 1), which causes an obvious decrease in the total binding free energy by about 2 kcal/mol (Table 1). H-bond analysis was performed based on the last 50 ns of MD trajectories. The number of H-bonds in the complex was larger for 6AP as compared to ADE. The H-bonds mainly involved three nucleotides U22, U51, and U74 (Figure 3A and Figure S2A, Supplementary Materials). The hydroxy of U22 formed stabilized H-bonds with the nitrogen atom in ADE and 6AP complexes. However, the polar contribution of solvation of U22 made up for the electrostatic contribution in the gas phase; therefore, the binding of U22 was mainly driven by van der Waals interactions (2.37 ± 0.07 kcal/mol) and nonpolar solvation energies. The amidogen of 6AP formed H-bonds with O2 atoms of U51 and U74, enhancing the electrostatic contribution in the gas phase in both GR(C74U) and AR complexes. The differences in electrostatic energies in complexes for U51 were 0.74 and 1.78 kcal/mol for ADE and 6AP, respectively. Similarly, the differences in energies for U74 were 1.91 and 1.77 kcal/mol for ADE and 6AP, respectively. Based on the crystallographic structures, we found that both ligands were located in the middle of two Watson–Crick base pairs: nucleotides A21–U75 and U22–A52 (Figure 3B and Figure S2B, Supplementary Materials). In the figures, we show the average distance between the mass center of the selected atoms of the nucleotide and the mass center of the ligand over the last 50 ns of MD trajectories obtained from 10 replica simulations. Therefore, nonpolar interactions due to parallel aromatic stacking represent the main contribution for nucleotides A21, U22, A52, and U75. As shown in Figure 3B and Figure S2B (Supplementary Materials), the ligands are stabilized in the binding pocket via an interaction with the surrounding nucleotides, implying that the presence of a ligand is very necessary to stabilize the binding pocket, thereby preventing an interaction between the side chains of nucleotides on both sides of the binding pocket. 

### 2.4. Comparison between 3AY and 3TT Complexes

**Figure 4:**
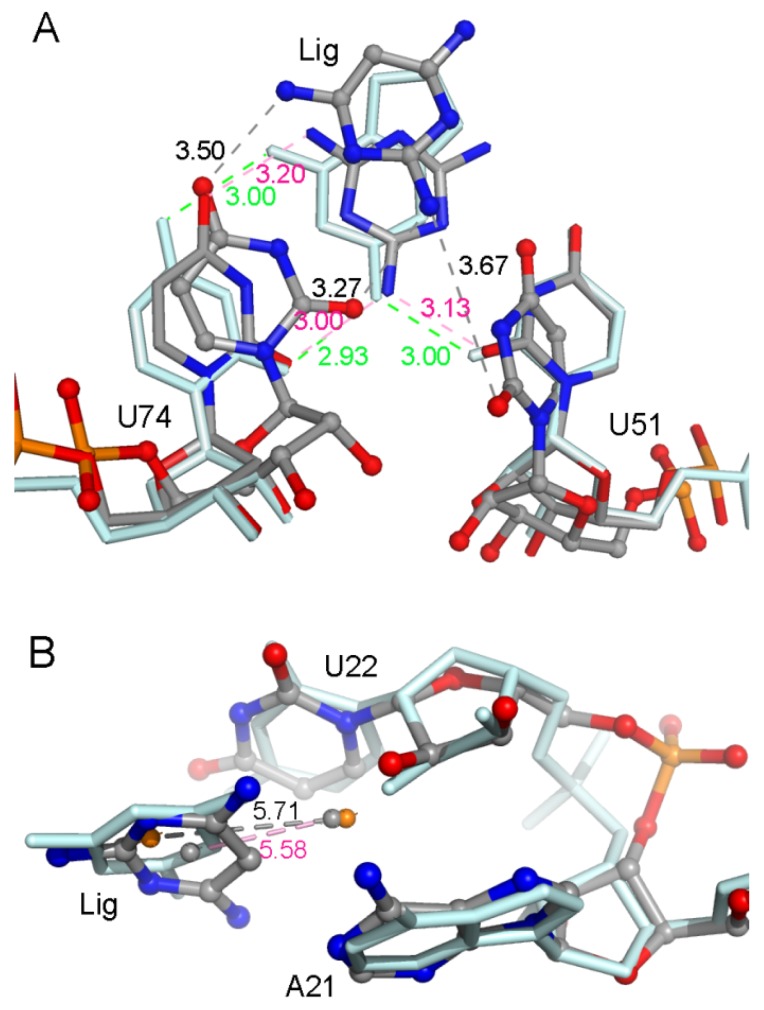
(**A**) Comparison of the H-bonds formed between ligands (6AP, 3AY, and 3TT) and nucleotides U51 and U74 in the GR(C74U) complexes; the representative structures are from the MD simulation. The distances of H-bonds are labeled in black, red, and green colors in 3AY, 3TT, and ADE, respectively. (**B**) The distances between the mass centers of 3AY/3TT and the two aromatic rings of A21 and U22 are labeled. The representative structures of ligands and nucleotides are from MD simulations; they are shown in stick-and-ball representation for the 3AY complex and in stick representation for the 3TT complex.

The atomic coordinates in the starting structures of 3AY and 3TT complexes were the same except for a nitrogen atom in 3TT corresponding to a carbon atom and a hydrogen atom in 3AY. The polar interaction was more unfavorable in 3AY than 3TT complexes; however, the nonpolar interaction was the opposite. The 3AY and 3TT structures were designed to mimic hypoxanthine, forming H-bonds with U51 and U74 [16]. According to the H-bond analysis, 3AY showed low occupancies in both GR(C74U) and AR complexes. Although the occupancies of 3TT were also low, its H-bonds were formed with different hydrogen atoms. However, 3TT had a higher occupancy as compared to 3AY for both complexes (Figure 4A and Figure S3A, Supplementary Materials), suggesting that 3TT is more inclined to imitate hypoxanthine than 3AY. Average RMSDs of the key six nucleotides of the ligand binding pocket were calculated (Table 2). We found that 3TT is more stabilized than 3AY in the binding pocket when we compared 3AY and 3TT. The probable reason for the stability of 3TT may be its close proximity to A21 and U22, as shown in Figure 4B and Figure S3B (Supplementary Materials).

### 2.5. Comparison between the Purine and Pyrimidine Analogues

Based on comparisons of the binding free energy spectra (Figure 2 and Figure S1, Supplementary Materials), we noticed that the binding free energies of nucleotide U74 were more favorable in purine analogues (ADE and 6AP) than in pyrimidine analogues (3AY, 3TT, and 2DY). The binding affinity of 6AP was larger than that of 3AY and 3TT (Table 1). Due to the lack of an imidazole ring, 3AY leaves the cavity very easily in the complexes compared to 6AP which has an imidazole ring. Similarly, 3TT in the complexes had a lower affinity due to the absence of the imidazole ring, which rendered the ligand and binding pocket more flexible. H-bond analysis indicated H-bonds between U50 and purine ligands; however, this type of H-bond was absent in the pyrimidine complex. As shown in Table 2, the average RMSDs of the key nucleotides for purine ligands were smaller than those for pyrimidine ligands. This may suggest a flexibility in the binding pocket for the pyrimidine ligands. Previously, we showed that the flexibility in the binding pocket can affect the global conformation [17]. 

### 2.6. The Dynamic Effects Caused by Ligand Binding

**Figure 5:**
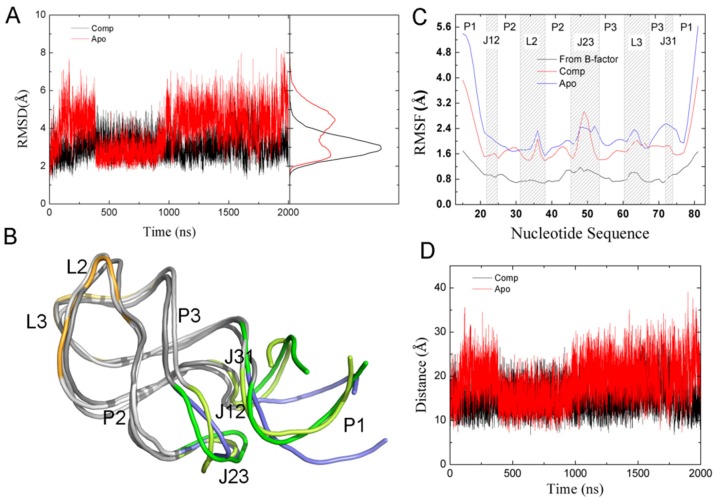
(**A**) The RMSDs and their distribution calculated on P, O3′, O5′, C3′, C4′, and C5′ atoms of the nucleotides of GR(C74U) relative to their starting structures. (**B**) The superimposition of two representative structures from Apo-GR(C74U) and one from the complex. All structures are shown in new cartoon representation. The P1J23 regions are shown in different colors. The open and closed conformations of Apo-GR(C74U) are in blue and yellow, while they are in green for the complex. (**C**) Root-mean-square fluctuations (RMSFs) for the P, O3′, O5’, C3′, C4′, and C5′ atoms of the nucleotides labeled in different regions. The experimental RMSFs from the B-factor are also given. (**D**) D1 versus the MD time for GR(C74U).

We carried out one 2-μs MD simulation each for apo-GR(C74U) and apo-AR, as well as in the presence of ADE for both complexes. RMSDs of heavy atoms were calculated relative to their starting structures as shown in Figure 5A and Figure S4A (Supplementary Materials). RMSDs for the complexes changed little during the 2-μs MD simulations. However, the RMSDs of Apo-GR(C74U) and Apo-AR increased clearly during the first 300 ns. A clear decrease in RMSD was found for the Apo-GR(C4U). Based on the distribution of RMSD values, two peaks were found for Apo-GR(C74U), and the other three systems had only one peak. This also suggests that Apo-GR(C74U) has two main conformations, which are interchangeable. Two structures taken from the two conformations were overlapped with the structure from the complex (Figure 5B). We found that one of the two conformations superimposed with the complex structure very well; however, the other was different from the complex structure in the P1L23 region. The variations from one conformation to the other were found at about 360 ns and 990 ns, which also suggests that the conformational changes for the purine riboswitch require at least 1 μs of MD simulation.

The root-mean-square fluctuations (RMSFs) of the P, O3′, O5′, C3′, C4′, and C5′ atoms of the nucleotides were calculated, as shown in Figure 5C and Figure S4B (Supplementary Materials). The experimental RMSFs were calculated from the X-ray temperature factors (*B*) using the equation $RMSF=\sqrt{3B/8\pi^{2}}$. The RMSFs of complexes and monomers were qualitatively similar to those from experimental data [16]. The RMSF values of most nucleotides were smaller in the complex than in the apo form. The experimental data and the calculated RMSFs for complexes and apo forms showed flexibility in the region P1J23 with a large RMSF value. This is consistent with the fact that GR promotes transcription through a stable anti-terminator stem in the absence of the ligand [4,25]. 

**Figure 6:**
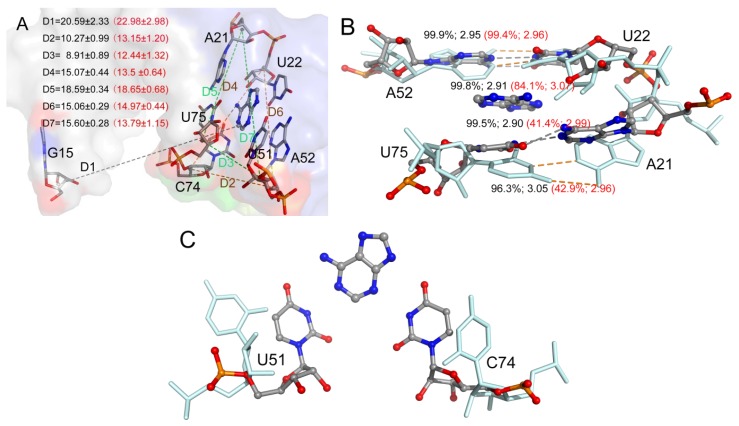
(**A**) Seven average distances labeling the binding pocket and conformational change between the mass centers of the backbone atoms of two nucleotides. The distance values with standard deviation are shown for the complex and monomer in black and red colors, respectively. (**B**) The H-bonds formed between key nucleotides in the binding pocket labeled with occupancies and average distances. (**C**) The position of two key nucleotides U51 and C74 in the complex and monomer.

To evaluate the conformation of the P1J23 region, we monitored the distance (D1) between the center of mass of nucleotide 1 and six key nucleotides, as shown in Figure 5D and Figure 6A. The average D1 distances calculated from the last 1 μs of total MD simulations for the complex and Apo-GR(C74U) were 20.59 and 22.98 Å, respectively. To quantitate the conformational state, we defined three states. When the D1 distance becomes greater than 22.98 Å, it forms the open state, and, when the distance becomes less than 20.59 Å, then it is in the closed state. Between the two states, there is a middle state. According to the definition, the percentages of closed and open states were 34.64% and 33.60% for Apo-GR(C74U), while, for the complex of GR(C74U), they were 55.39% and 12.72%. Therefore, the complex is more favorable in the closed state compared to the Apo aptamer.

We also monitored six key distances (D2–D7) in the binding pocket which could reflect the conformational changes (Figure 6A). As shown in Figure 6, the distances D2 and D3 were larger in the complex than Apo-GR(C74U); however, D4 and D7 were smaller, while D5 and D6 changed slightly. Thus, the pocket with the ligand (complex) was obviously different from that without the ligand (Apo). The two Watson–Crick base pairs were stabilized in the complex with a stronger H-bond interaction (Figure 6B), having smaller distances and larger occupancies relative to Apo-GR(C74U). The other two key nucleotides (U51 and C74) located in the binding pocket showed different conformations in the complex relative to Apo-GR(C74U) (Figure 6C). 

### 2.7. The Relationship between Conformational Change and Ligand Binding

The long MD simulations of GR(C74U) showed that the conformations of Apo-GR(C74U) shifted between closed and open states, and the complex was mainly in the closed state. To calculate binding free energies, we performed 10 unbiased MD simulations for 100 ns each for the individual complexes with different starting conditions. In general, MD simulations with different initial conditions result in slightly different values for system properties [53]. Therefore, we analyzed 50 100-ns MD trajectories each for the GR(C74U) complexes and AR complexes. The percentages of structures in the open and closed states were 17.6% and 48.7% for GR(C74U) complexes, which were nearly similar to those for AR complexes with values of 19.8% and 46.3% for open and closed states, respectively. In 10 of the 50 MD simulations, the open state predominated over the closed state for GR(C74U) complexes, reflecting that one state can shift to another state even in the presence of a ligand. 

**Figure 7:**
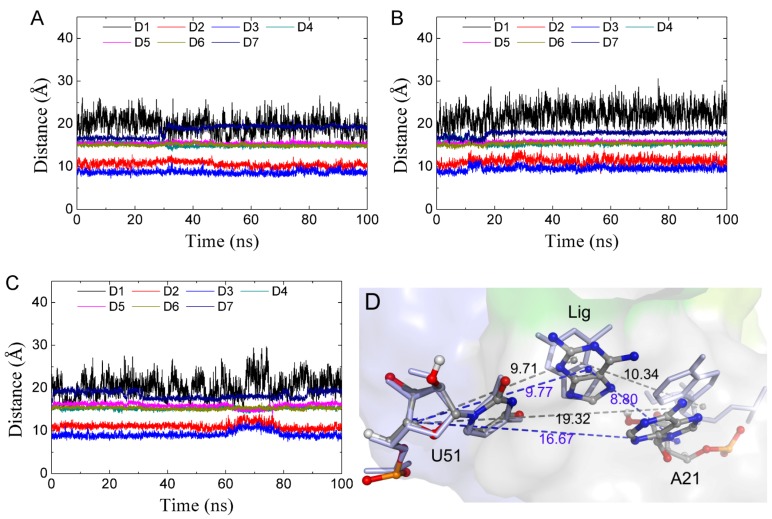
The key distances described in Figure 6 versus MD simulation time. (**A**) Closed state. (**B**) Conformational change at the start of the MD simulation. (**C**) Open state in the middle of the MD simulation. (**D**) The position of A21 for two representative structures of D7.

In order to investigate the relationship between the conformation of the ligand binding pocket and the conformational state, we monitored key distances in the binding pocket, as shown in Figure 6A for the complexes. The results show that distances were stabilized with the system in the closed state except for D7 (Figure 7A). Next, we focused on the trajectories of the 50 MD simulations in which the state changed significantly. There were six MD simulations having the conformation switch from a closed to open state, which was observed at the beginning of the MD simulation (Figure 7B and Figure S5, Supplementary Materials). At this transition (closed to open) period, was a significant increase in the D2 and D3 distances. We found a short open state in the middle of the trajectory from one MD simulation (Figure 7C), in which a short increase in D2 and D3 was observed. We calculated the average distances between the mass center of U51 and A52 and the mass center of C74 and C75 in each complex, as shown in Table 3. Table 3 also shows the percentages of the closed and open states for each ligand. The correlation coefficients of the distances with percentages of states were 0.66 and 0.72 for GR(C74U) and AR complexes, respectively, for the open state. The distances showed a negative correlation with the percentage in a closed state. Thus, the conformational state may be related to distances D2 and D3. When D2 and D3 become small, the conformation remains in a closed state, and vice versa. We also noted that the complexes of 3TT showed the largest percentage of the closed state in both the GR(C74U) and the AR complexes; however, its binding affinity was not the strongest (Table 1). This result is in accordance with the finding that binding affinity is not the sole parameter that governs the activity of RNA-binding compounds [54,55]. Hence, it is a favorable strategy to take the dynamic characteristics of the binding pocket into account to design new ligands for adenine riboswitches in the future, which further suggests that the dynamic characteristics of the binding pocket are very useful and may be beneficial for designing new ligands in the future.

Among the 50 MD simulations, 35 MD simulations were found with a significant change in D7 with an average of 16.7 to 19.3 Å. The variation was found at the beginning, at the middle, and at the end of the 100-ns MD simulations, suggesting that the variation in D7 is a general phenomenon. The 15 100-ns MD simulations without variation required longer MD time. As shown in Figure 7D, the variation in D7 was mainly caused by A21, resulting in an increase in the distance of A21 from the ligand. 

## 3. Materials and Methods

### 3.1. System Preparation

The starting structures of MD simulations were retrieved from the Research Collaboratory for Structural Bioinformatics (RCSB) Protein Data Bank (PDB). The PDB identifier (ID) for the GR(C74U) and 3AY complexes is 2G9C [16], and that for the AR and ADE complexes is 1Y26 [15]. All crystallographic water molecules were retained. The ligand in the binding pocket of GR(C74U) or AR was replaced with the new ligand to obtain the new complex. All complexes were solvated in a rectangular periodic box of Transferable Intermolecular Potential 3 Point (TIP3P) [56] water molecules with a margin distance of 12 Å. An appropriate number of sodium counterions were added to neutralize the system charges. The missing parameters of ligands were developed. Single-point calculations were performed with Gaussian 03 [57] for each ligand using the Hartree–Fock/6-31G* basis set to obtain the electrostatic potential, which was used to assign the partial charges for each atom using the Restrained Electrostatic Potential (RESP) method [58] with the antechamber module of the AMBER12 package. The general AMBER force-field (GAFF) parameters [59] were used to describe the ligands. The parameters of RNA, ions, and water molecules were described by the AMBER force field (FF12SB) [60]. 

### 3.2. Molecular Dynamics Simulation

All energy minimizations and MD simulations were carried out using the AMBER14 package [61]. The systems were minimized by 50 steps of steepest-descent minimization followed by 200 steps of conjugate-gradient minimization with a force constant of 25 kcal/(mol·Å^2^) harmonic restraints to all solute atoms. Then, the same minimization was performed again except for a force constant of 5 kcal/(mol·Å^2^). After minimization, the systems were heated from 100 K to 300 K over 40 ps for a constant-volume MD simulation, then over 10 ps at 300 K. Subsequently, a constant-pressure MD simulation was carried out for 50 ps to adjust the solvent density. Then, we carried out six 50-ps constant-volume MD simulations with force constants from 5 to 0 kcal/(mol·Å^2^) harmonic restraints. Finally, the production constant-pressure MD simulations without any restraints were performed at 300 K with a target pressure of 1.0 atm. The SHAKE algorithm was used to treat the covalent bonds involving hydrogen atoms [62]. The particle mesh Ewald method was applied to treat long-range electrostatic interactions [63]. The time step was set to 2 fs for all MD simulations. For each complex, 10 unbiased simulations were performed for 100 ns each. The long MD simulations for the monomers and its complexes with ADE were run for 2 μs. Coordinate trajectories were recorded every 10 ps throughout all production runs.

### 3.3. Binding Free Energy Calculations

For each complex, the binding free energies ($\Delta G_{\mathrm{bind}}$) were estimated based on 10 stabilized 100-ns MD trajectories using the MM-PBSA method. For each trajectory, we extracted 500 snapshots from the last 50 ns of the MD trajectories with an interval of 100 ps. Briefly, the MM-PBSA method was used to calculate the binding free energies using the following equations [64,65]:

(1) $$\Delta G_{\mathrm{bind}}=\;\Delta H_{\mathrm{gas}}+\Delta G_{\mathrm{solv}}-T\Delta S,$$

(2) $$\Delta H_{\mathrm{gas}}=\Delta E_{\mathrm{int}}+\Delta E_{\mathrm{vdW}}+\Delta E_{\mathrm{ele}},$$

(3) $$\Delta G_{\mathrm{solv}}=\Delta G_{\mathrm{pol}}+\Delta G_{\mathrm{nonpol}},$$

Where $\Delta H_{\mathrm{gas}}$ is the total molecular mechanical energy in the gas phase, and it is the sum of internal energy ($\Delta E_{\mathrm{int}}$), Van der Waals energy ($\Delta E_{\mathrm{vdw}}$), and the electrostatic energy ($\Delta E_{\mathrm{ele}}$) in the gas phase. The solvation free energy ($\Delta G_{\mathrm{solv}}$) is divided into the polar ($\Delta G_{\mathrm{pol}}$) and nonpolar ($\Delta G_{\mathrm{nonpol}}$) components calculated by using the PB/SA model. The $\Delta G_{\mathrm{pol}}$ was calculated by numerically solving the Poisson Boltzmann equation. The $\Delta G_{\mathrm{nonpol}}$ owing to cavity formation and van der Waals interactions between the solute and solvent was estimated by the solvent-accessible surface area (SASA) with $\Delta G_{\mathrm{nonpol}}=\;\gamma SASA+\beta$ [66]. The normal mode analysis was used to obtain the conformational entropic contribution [67].

## 4. Conclusions

The ligand binding and conformational transition mechanisms were using by MD simulations, as well as binding free energy calculations. The binding free energies obtained using the MM-PBSA method are in agreement with the experimental data. We further found that the ligands bind to GR(C74U) and AR with the same binding model; however, the electrostatic energies are more unfavorable for all ligands in GR(C74U) compared to AR complexes. The comparison between two pyrimidine analogues revealed that a nitrogen atom at the 5-position is more favorable than a carbon atom to mimic the natural ligand of AR. Four 2-μs MD simulations suggest that the conformations are in three states (open, middle, and closed). The complexes are mainly in the closed state. The Apo-GR(C75U) shifts its conformation between open and closed states. The conformational state is associated with the distance between A52 and C74, as well as the distance between U51 and U75, which can provide insight for designing new ligands.

## Figures and Tables

**Table 1:** Binding free energies calculated for GR(C74U) and AR complexes using the molecular mechanics Poisson–Boltzmann surface area (MM-PBSA) method ^a^.

Items ^b^	ADE		6AP		3AY		3TT		2DY	
	GR(C74U)	AR	GR(C74U)	AR	GR(C74U)	AR	GR(C74U)	AR	GR(C74U)	AR
${\Delta E}_{\mathrm{ele}}$	−31.48 ± 0.45	−31.67 ± 0.61	−34.35 ± 0.90	−36.87 ± 0.89	−30.77 ± 0.76	−29.49 ± 0.55	−32.23 ± 1.09	−32.10 ± 0.49	−25.57 ± 1.49	−24.71 ± 1.13
${\Delta E}_{\mathrm{vdw}}$	−25.45 ± 0.28	−25.98 ± 0.40	−27.56 ± 0.32	−27.49 ± 0.38	−23.55 ± 0.42	−23.21 ± 0.47	−22.90 ± 0.48	−22.48 ± 0.54	−20.93 ± 0.44	−20.43 ± 0.44
${\Delta E}_{\mathrm{int}}$	1.42 ± 0.02	1.48 ± 0.02	1.39 ± 0.02	1.50 ± 0.02	1.39 ± 0.02	1.48 ± 0.02	1.40 ± 0.01	1.45 ± 0.02	1.37 ± 0.02	1.51 ± 0.02
${\Delta G}_{\mathrm{nonpol}}$	−2.86 ± 0.02	−2.84 ± 0.02	−3.01 ± 0.02	−3.00 ± 0.02	−2.81 ± 0.01	−2.81 ± 0.01	−2.75 ± 0.02	−2.76 ± 0.01	−2.70 ± 0.01	−2.73 ± 0.01
${\Delta G}_{\mathrm{pol}}$	35.54 ± 0.72	33.64 ± 0.91	36.35 ± 0.01	36.45 ± 0.01	34.63 ± 1.41	32.31 ± 1.17	34.88 ± 1.50	32.89 ± 1.06	29.52 ± 1.55	28.74 ± 0.87
${\Delta G}_{\mathrm{ele}+\mathrm{pol}}$	4.06 ± 0.46	1.97 ± 0.43	2.00 ± 1.15	−0.42 ± 0.89	3.85 ± 1.25	2.81 ± 1.17	2.66 ± 1.60	0.80 ± 1.13	3.96 ± 1.23	4.03 ± 0.93
${\Delta G}_{\mathrm{vdw}+\mathrm{nonpol}}$	−28.30 ± 0.28	−28.83 ± 0.39	−30.57 ± 0.65	−30.48 ± 0.18	−26.36 ± 0.42	−26.01 ± 0.47	−25.64 ± 0.47	−25.23 ± 0.54	−23.63 ± 0.43	−23.17 ± 0.43
ΔH	−22.83 ± 0.62	−25.38 ± 0.57	−27.18 ± 0.31	−29.40 ± 0.38	−21.12 ± 1.42	−21.72 ± 1.18	−21.59 ± 1.90	−22.98 ± 1.18	−18.31 ± 1.06	−17.63 ± 1.10
TΔS	−13.31 ± 0.24	−13.62 ± 0.31	−14.58 ± 0.79	−14.03 ± 0.46	−14.69 ± 0.33	−14.46 ± 0.28	−15.29 ± 0.20	−15.30 ± 0.22	−13.72 ± 0.25	−13.22 ± 0.22
${\Delta G}_{\mathrm{bind}}$	−9.53 ± 0.73	−11.76 ± 0.41	−12.60 ± 0.40	−15.37 ± 0.31	−6.42 ± 1.55	−7.27 ± 0.98	−6.30 ± 1.86	−7.68 ± 1.10	−4.58 ± 1.21	−4.41 ± 1.14
${\Delta G}_{\exp}$ ^c^	−8.78 (−8.8)	−9.20 (−9.2)	−10.57 (−11)	−11.53 (−12)	−6.45 (−6.6)	−7.82 (−7.8)	−6.45 (−6.5)	null	null	null

^a^ The unit is kcal/mol. Standard errors denoted with ± signs were calculated by *σ* = standard deviation/*N*1/2, where *N* is 10 for the 10 independent molecular dynamics (MD) simulations for each complex. ^b^ The symbols of the energy terms are the same as in the section of the binding free energy calculations. ^c^ The experimental values are from References [16,26] calculated from $\Delta G_{\exp}=-RT\ln K_{d}$; the values from the references are shown in round brackets.

**Table 2:** The average root-mean-square deviations (RMSDs) of the key six nucleotides (Å).

Items	ADE	6AP	3AY	3TT	2DY
GR(C74U)	1.03 ± 0.37	1.27 ± 0.32	1.41 ± 0.58	1.23 ± 0.35	1.49 ± 0.46
AR	1.12 ± 0.33	1.04 ± 0.24	1.41 ± 0.39	1.32 ± 0.33	1.34 ± 0.46

**Table 3:** The percentages of conformational states and the distances (Å).

Items	GR(C74U)			AR		
	Open	Closed	Distance ^a^	Open	Closed	Distance ^a^
ADE	19.82%	43.22%	10.08	19.82%	43.22%	10.08
6AP	18.33%	48.49%	10.00	23.83%	39.74%	10.07
3AY	19.44%	49.12%	10.25	15.88%	52.27%	10.14
3TT	11.76%	56.64%	9.89	13.26%	55.36%	10.06
2DY	18.69%	46.28%	10.38	28.23%	35.44%	10.59
*R* ^b^	0.66	−0.56		0.72	−0.62	

^a^ The distances between the mass center of U51 and A52 and the mass center of C74 and C75. ^b^ The correlation coefficients are for the conformational states and the distances.

## Supplementary Materials

Supplementary materials can be found at https://www.mdpi.com/1422-0067/21/6/1926/s1. Figure S1: The decomposition of inhibitors on a per-nucleotide basis. Figure S2: The same as in Figure 3 except for the RNA is the AR. Figure S3: The same as in Figure 4 except for the RNA is the AR. Figure S4: (A) The RMSDs of apo and comp calculated on P, O3’, O5’, C3’, C4’, and C5’ atoms of the nucleotides of AR relative to their starting structures. (B) RMSFs for the P, O3’, O5’, C3’, C4’, and C5’ atoms of the nucleotides. The experimental RMSFs from the B-factor are also given. Figure S5: The key distances describe in Figure 6 versus the MD simulation time for conformational state change at the starting of the MD simulation stage.
