# Supplementary material for: Ligand Binding Mechanism and Its Relationship with Conformational Changes in Adenine Riboswitch
Source: Int J Mol Sci. 2020 Mar 11;21(6):1926. doi: 10.3390/ijms21061926 (PMC7139962; doi:10.3390/ijms21061926)
Supplement: Supplementary file 1 [file ijms-21-01926-s001.pdf]

Ligand Binding Mechanism and Its Relationship with Conformational Changes in Adenine Riboswitch

Guodong Hu*, Haiyan Li, Shicai Xu and Jihua Wang*

Shandong Key Laboratory of Biophysics, Dezhou University, Dezhou 253023, China;

tianwaifeixian78@163.com(Y.L.); shicaixu@dzu.edu.cn(S.X.)

* Correspondence: E-Mails:xzszhgd@163.com (G. H.); jhw25336@126.com (J.W.); Tel.:

+86-534-8987536 (G. H.); +86-534-8985933 (J.W.)

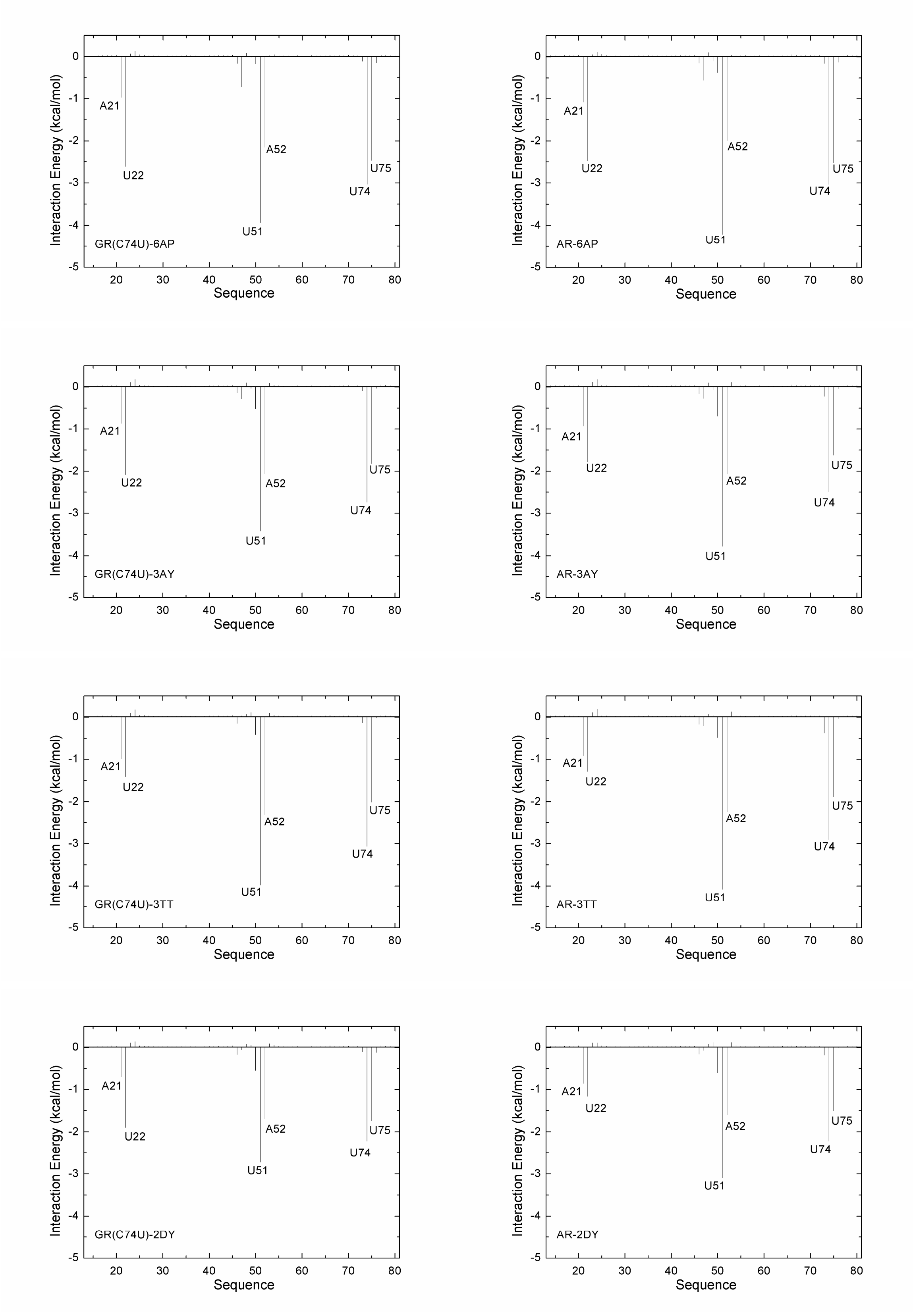

Figure S1 The decomposition of inhibitors on a per-nucleotide basis.

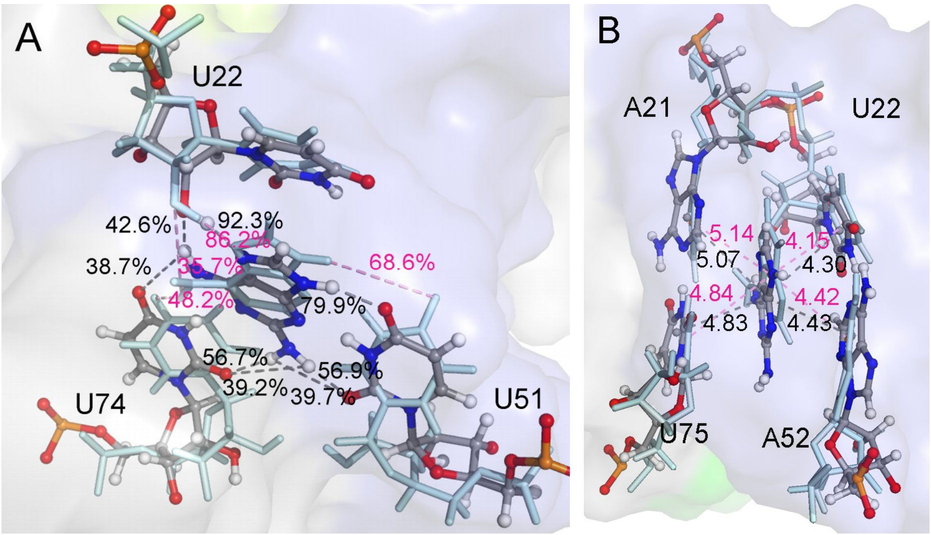

Figure S2 The same as in Fig. 3 except for the RNA is the AR.

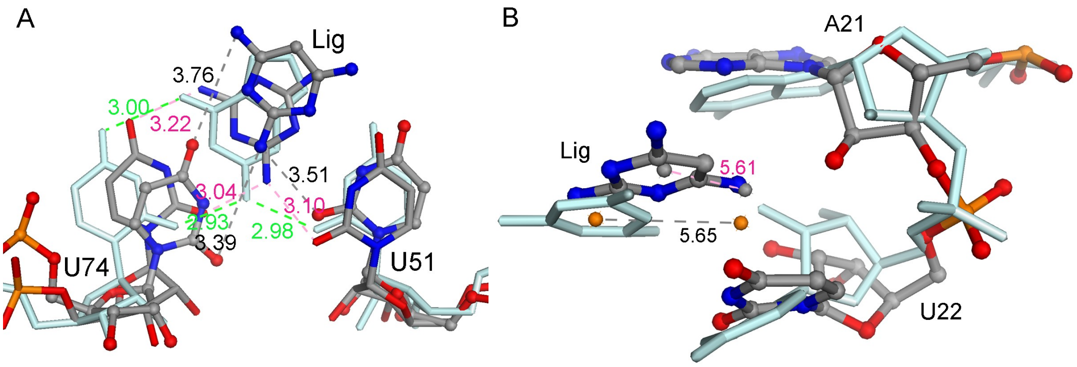

Figure S3 The same as in Fig. 4 except for the RNA is the AR.

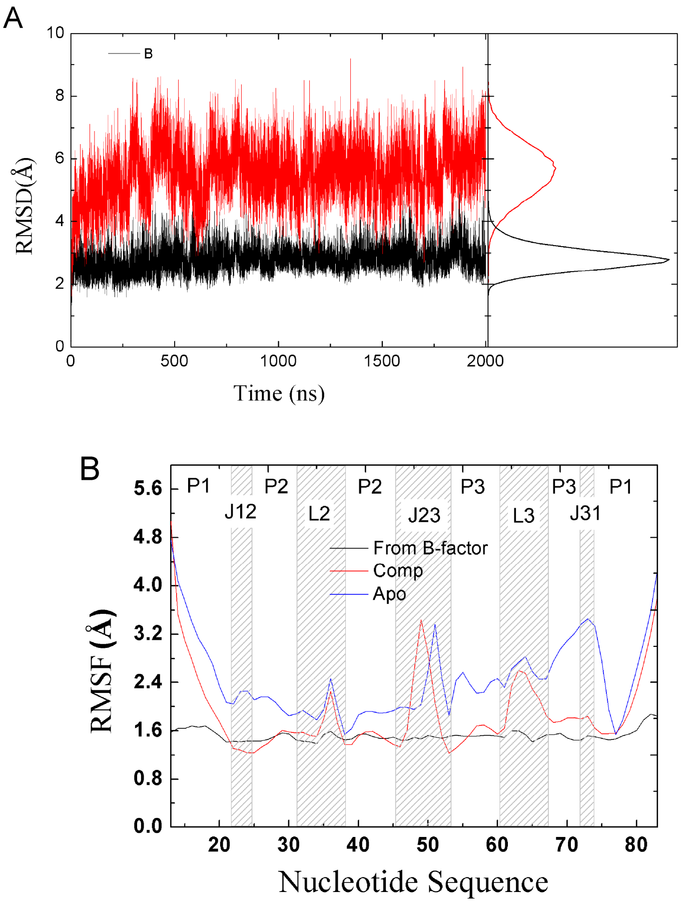

Figure S4 (A) The RMSDs of apo and comp calculated on P, O3', O5', C3', C4', and C5' atoms of the nucleotides of AR relative to their starting structures. (B) RMSFs for the P, O3', O5', C3', C4', and C5' atoms of the nucleotides. The experimental RMSFs from the B-factor are also given.

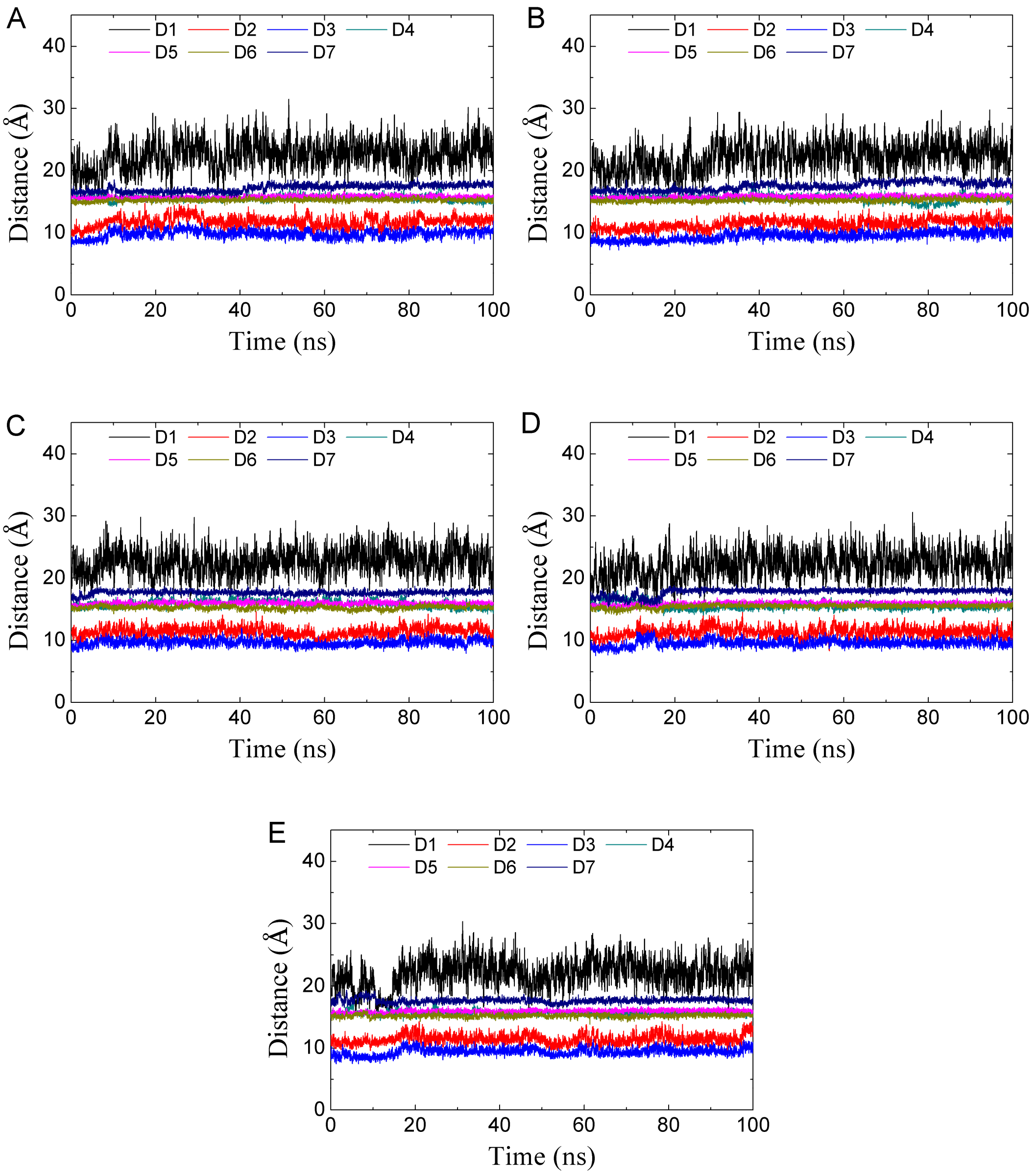

Figure S5 The key distances describe in Fig. 6 versus the MD simulation time for conformational state change at the starting of the MD simulation stage.
